# High level of genomic divergence in *orf-I* p12 and *hbz* genes of HTLV-1 subtype-C in Central Australia

**DOI:** 10.1186/s12977-024-00647-w

**Published:** 2024-07-17

**Authors:** Ashley Hirons, David Yurick, Natasha Jansz, Paula Ellenberg, Genoveffa Franchini, Lloyd Einsiedel, Georges Khoury, Damian F. J. Purcell

**Affiliations:** 1 The Peter Doherty Institute for Infection and Immunity, Department of Microbiology and Immunology, The University of Melbourne, Melbourne, VIC Australia; 2 Mater Research Institute-University of Queensland, TRI Building, Woolloongabba, QLD Australia; 3 Animal Models and Retroviral Vaccines Section, National Cancer Institute, National Institutes of Health, Bethesda, MD USA; 4 Department of Medicine, Alice Springs Hospital, Alice Springs, NT Australia; 5 Present Address: UCB Pharma, Smyrna, GA USA; 6 Present Address: Burnet Institute, Melbourne, VIC Australia; 7 Present Address: Kite Pharma, Santa Monica, CA USA

## Abstract

**Background:**

Human T cell lymphotropic virus type 1 (HTLV-1) infection remains a largely neglected public health problem, particularly in resource-poor areas with high burden of communicable and non-communicable diseases, such as some remote populations in Central Australia where an estimated 37% of adults are infected with HTLV-1. Most of our understanding of HTLV-1 infection comes from studies of the globally spread subtype-A (HTLV-1a), with few molecular studies reported with the Austral-Melanesian subtype-C (HTLV-1c) predominant in the Indo-Pacific and Oceania regions.

**Results:**

Using a primer walking strategy and direct sequencing, we constructed HTLV-1c genomic consensus sequences from 22 First Nations participants living with HTLV-1c in Central Australia. Phylogenetic and pairwise analysis of this subtype-C proviral gDNA showed higher levels of genomic divergence in comparison to previously published HTLV-1a genomes. While the overall genomic homology between subtypes was 92.5%, the lowest nucleotide and amino acid sequence identity occurred near the 3′ end of the proviral genome coding regulatory genes, especially overlapping *hbz* (85.37%, 77.46%, respectively) and *orf-I* product p12 (82.00%, 70.30%, respectively). Strikingly, the HTLV-1c genomic consensus sequences uniformly showed a defective translation start codon for the immune regulatory proteins p12/p8 encoded by the HTLV-1A *orf-I*. Deletions in the proviral genome were detected in many subjects, particularly in the structural *gag*, *pol* and *env* genes. Similarly, using a droplet digital PCR assay measuring the copies of *gag* and *tax* per reference host genome, we quantitatively confirmed that provirus retains the *tax* gene region at higher levels than *gag*.

**Conclusions:**

Our genomic analysis of HTLV-1c in Central Australia in conjunction with earlier Melanesian HTLV-1c sequences, elucidate substantial differences with respect to the globally spread HTLV-1a. Future studies should address the impact these genomic differences have on infection and the regionally distinctive frequency of associated pulmonary disease. Understanding the host and virus subtype factors which contribute to the differential morbidity observed, is crucial for the development of much needed therapeutics and vaccine strategies against this highly endemic infection in remote First Nations communities in Central Australia.

## Background

An estimated 10–20 million individuals worldwide are infected with human T cell lymphotropic virus type 1 (HTLV-1) [1]. Unfortunately, low awareness and low testing rates for this lifelong infection among communities in high risk endemic hotspots, such as Central and Western Africa [2] and First Nations communities in Central Australia [3], obscures the true global health burden of HTLV-1. A minority of people infected with HTLV-1 develop either HTLV-1 associated myelopathy (HAM) [4], a chronic inflammatory condition resulting in spinal cord injury, or adult T cell leukemia/lymphoma (ATL), an aggressive malignancy of CD4 + T-cells with very poor prognosis [5, 6]. Nonetheless, a recent meta-analysis found a 60% increase in mortality among people with HTLV-1 that was not accounted for by HAM or ATL [7]. Less well recognised HTLV-1 associated inflammatory diseases include HTLV-1 associated pulmonary disease (HAPD), which typically presents as bronchiolitis and may progress to bronchiectasis [8]. HAPD was first reported in Japan [9], but is well documented in Central Australia [10–12], where blood stream infections (BSIs) [13, 14], and two non-communicable diseases, chronic kidney disease (CKD) and diabetes [15, 16], also disproportionately affect people living with higher HTLV-1 subtype-C (HTLV-1c) proviral load (PVL) at, or above, 1% of peripheral blood mononuclear cells (PBMC).

HTLV-1 integrates a copy of reverse transcribed DNA into the host genome (provirus) during successful infection of T-cells, and this modulates host cell phenotype and function to achieve lifetime persistence in the host [17]. HTLV-1’s ability to avoid immune detection while maintaining or increasing the proviral reservoir provides an obstacle to treatments and preventions. Halting the spread of the virus in resource-poor communities has been difficult, as no licenced pre-exposure prophylactics nor effective vaccines exist at present [17]. Moreover, there are no directly acting antiviral therapies developed for targeting this lifelong and complex retroviral infection.

PVL is the main correlate for progression of HTLV-1 associated diseases, both worldwide [18–20] and in Central Australia, where a cohort follow up revealed donors with higher HTLV-1c PVL ≥ 1% PBMC had significantly lower life expectancy [12, 14]. The molecular mechanisms exploited by this oncoretrovirus to facilitate viral persistence in the human host by maintaining or increasing the viral reservoir include the multifaceted functions of regulatory genes and proteins such as Tax, *hbz/*HBZ, and *orf-I*/p12/p8 that are encoded towards the 3′ end of the viral genome, historically termed the *pX*-region [21–26]. However, most of our understanding of this infection has been achieved through studying the cosmopolitan HTLV-1 subtype-A (HTLV-1a).

Unfortunately, our understanding of HTLV-1c, endemic in Central Australia and surrounding islands in Oceania, is largely based on partial genome sequences of long terminal repeats (LTR) and envelope (env), while currently only five single complete HTLV-1c genomic sequences (MEL5, Aus-CS, Aus-DF, Aus-NR, Aus-GM) have been assembled and published [3, 27]. In this study, we examined integrated proviral genomic DNA of 22 HTLV-1c infected participants from a remote Central Australian setting. We performed comparative genomic and phylogenetic analysis that revealed novel key mutations and genetic differences of subtype-C compared to the globally spread subtype-A. In addition, we observed proviral deletions in the majority of participants, particularly in the structural genes.

## Results

The paucity of community-based data leaves large gaps in our understanding of HTLV-1c infection and its prevalence and pathogenesis within Central Australian First Nations communities. To better understand the similarity of HTLV-1c strains regionally and differences with the globally dispersed HTLV-1a, thirty-seven individuals were recruited to participate in this study at the Alice Spring Hospital (ASH) that serves remote communities over 10^6^ square kilometres. Participants included 22 HTLV-1c positive participants and 15 uninfected subjects with a median age of 49.5 and 40.0 years, respectively (p 0.15). A summary of the demographic data for the 37 participants is listed in Table 1. No significant difference in the sex assigned at birth distribution was observed (p 0.52). The single clinical characteristic which distinguished the HTLV-1c positive subjects in this cohort was a significantly higher level of blood stream infections (BSIs) with common bacterial species in 10 out of 22 infected individuals, while no BSI events were recorded for the uninfected participants (p 0.002), similar to previous reports in this population [13]. We did not observe any difference in the frequency of pulmonary disease, encompassing bronchiectasis, bronchiolitis, chronic suppurative lung disease (CSLD) and chronic obstructive pulmonary disease (COPD), between HTLV-1c infected and uninfected participants (p 0.74). However, HAPD is well documented in the region with larger scale clinical and epidemiological reports [8, 11, 12, 28, 29].

**Table 1:** Demographics and clinical characteristics of participants from Central Australia according to HTLV-1c status

	HTLV-1c– (n = 15)	HTLV-1c + (n = 22)	p-value
Demographics, n (%)			
Median age	40.0	49.5	0.15
Female at birth	7/15 (46.7%)	13/22 (59.1%)	0.52
Male at birth	8/15 (53.3%)	9/22(40.1%)	
Lifestyle, n (%)			
Smoker (current or previous)	8/15 (53.3%)	9/15 (60.0%)^Ø^	> 0.99
Harmful alcohol consumption	9/15 (60.0%)	9/17 (52.9%)^Ø^	0.73
Comorbidities, n (%)			
Asthma	2/15 (13.3%)	2/22 (9.1%)	> 0.99
Ischaemic heart disease	2/15 (13.3%)	4/22 (18.2%)	> 0.99
Diabetes	6/15 (40.0%)	11/22 (50%)	0.72
Chronic liver disease (CLD)	0/15 (0%)	3/22 (13.6%)	0.26
Chronic kidney disease (CKD)	7/15 (46.7%)	13/22 (59.1%)	0.52
Hepatitis B Virus (HbsAg)	0/9 (0%)^Ø^	2/20 (10.0%)^Ø^	> 0.99
Potential HTLV-1 associated diseases, n (%)			
Pulmonary Disease	5/15 (33.3%)	9/22 (40.1%)	0.74
Strongyloidiasis, current	2/15 (13.3%)	2/21 (9.5%)^Ø^	> 0.99
Blood stream infection (BSI)	0/15 (0%)	10/22 (45.5%)	0.002**
HTLV-1 associated myelopathy (HAM)	0/15 (0%)	0/22 (0%)	> 0.99
Adult T cell leukemia/lymphoma (ATL)	0/15 (0%)	0/22 (0%)	> 0.99

** Bold indicates high statistical significance, p ≼ 0.01

HbsAg, HBV surface antigen

BSI includes current or previous infection

Pulmonary disease includes chronic obstructive pulmonary disease (COPD), chronic suppurative lung disease (CSLD), bronchiectasis and bronchiolitis

Missing data are indicated with Ø

Statistical significance assessed using Mann–Whitney test for continuous variables and Fisher’s exact test for categorical variables

### People living with HTLV-1c predominantly carry defective provirus

**Fig. 1:**
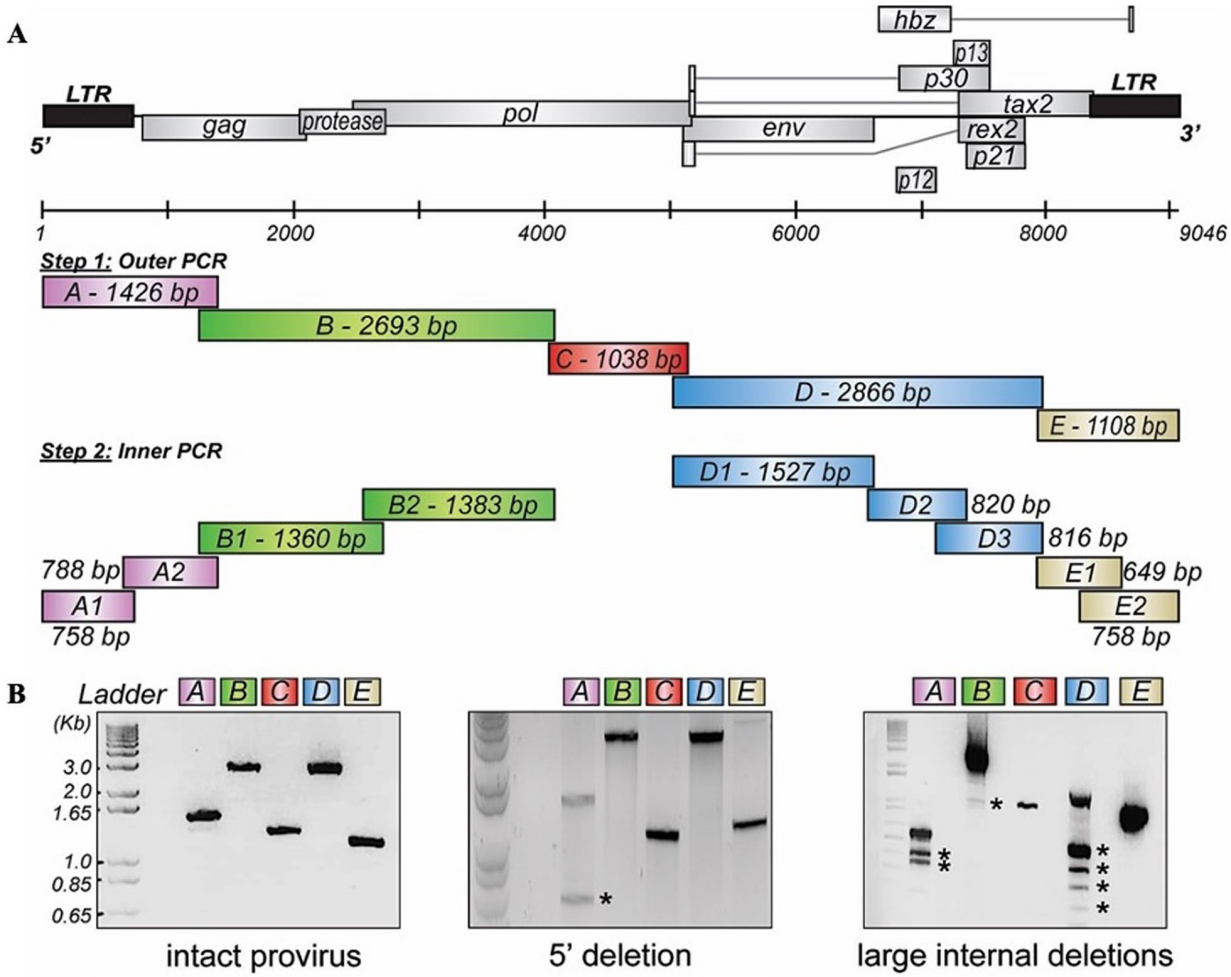
Overlapping fragment PCR strategy to assemble whole HTLV-1c genomes. **A** An overlapping PCR strategy was used to amplify the complete 9 kb HTLV-1c proviral genome from gDNA isolated from 22 infected participants. Five sets of oligonucleotides were used, each amplifying a region of the HTLV-1c genome: A (odp3100-odp3160, 1426nt); B (odp3161-odp3162, 2693nt); C (odp3163-odp3164, 1038nt); D (odp3165-odp3141, 2866nt) and E (odp3166-odp3101, 1108nt). The outer PCR fragments were then subjected to a nested PCR using overlapping primer pairs, followed by direct sequencing of the purified PCR products and assembly of the reads. **B** Representative agarose gel electrophoreses of PCR amplicons show intact sequences (MT2 cell line gDNA), a 5′ deletion and large internal deletions (HTLV-1c + gDNA). Asterisks (*) indicate amplicons with a smaller size than expected, which is likely derived from deleted proviruses

Unique nucleotide features may contribute to the distinctive pulmonary disease pathogenesis and progression reported for HTLV-1c [8, 10–14, 28]. To faithfully characterize the entire HTLV-1c proviral genome, genomic DNA samples isolated from the PBMC of 22 HTLV-1c + participants were subjected to a genome walking strategy [3], which consists of five series of PCRs using primers (Table 2) targeting conserved regions within the HTLV-1 genome. The five genomic regions (A, 5′LTR-*gag*; B, *gag*-*pol*; C, *pol*; D, *env*-3′ regulatory region; E, 3′regulatory region-3′LTR) were then subjected to nested PCRs (Fig. 1A). Agarose gel electrophoresis of subgenomic PCRs revealed that most participants (21 out of 22) may carry proviral genomic deletions, as indicated by amplicons of reduced size in addition to, or instead of, the expected DNA fragment, in the 5′LTR region and in the internal structural and regulatory coding regions (Fig. 1B). The amplified products at expected sizes were purified and sequenced by Sanger method on both strands using a series of forward and reverse primers (Table 2).

**Table 2:** Primers and probes used in this study

ODP ID	Sequence (5′ → 3′)	Purpose
HTLV-1c PVL quantification by ddPCR		
3083	CAAATGAAGGACCTACAGGC	Forward primer for HTLV-1c *gag*
3084	TATCTAGCTGCTGGTGATGG	Reverse primer for HTLV-1c *gag*
3321	6FAM-ACCATCCGGCTTGCAGT-MGBNFQ	Probe for HTLV-1c *gag*
3085	TCCAGGCCTTATTTGGACAT	Forward primer for HTLV-1c *tax*
3086	CGTGTGAGAGTAGGACTGAG	Reverse primer for HTLV-1c *tax*
3318	6FAM-CATGATTTCCGGGCCTTGC-MGBNFQ	Probe for HTLV-1c *tax*
Unrearranged T-cell receptor beta (UTCRβ) locus quantification by ddPCR		
3190	TGTACAAAGCTGTAACATTGTGGGGAC	Forward primer for UTCRβ
3191	AACCAAATTGCATTAAGACCTGTGACC	Reverse primer for UTCRβ
3191a	6FAM-ACAATGATTCAACTCTACGGGAAACC-MGBNFQ	Probe for UTCRβ
Primer walking strategy, step 1—outer PCR		
3100	ATAAACTTAAGTGATACTGACCATGGGCCCCAAATAC	Forward primer for fragment A
3160	CTATCTAGCTGCTGGTGATGGAGGG	Reverse primer for fragment A
3161	CCCTCCATCACCAGCAGCTAGATAG	Forward primer for fragment B
3162	GAGTCTAAAAATATGTTGAGACAGTGCC	Reverse primer for fragment B
3163	GGCACTGTCTCAACATATTTTTAGACTC	Forward primer for fragment C
3164	ATAAAGGATCCACTGGGCAGAATTGGCGC	Reverse primer for fragment C
3165	ATAAAGGATCCCGTGGAGGCTCCTCAAGC	Forward primer for fragment D
3141	GGAAAAGGGTGGTGGGTAAACAGCC	Reverse primer for fragment D
3166	GGCTGTTTACCCACCACCCTTTTCC	Forward primer for fragment E
3101	ATAAACTTAAGTGTGTTCTATGTCTCTCTCCTGGAGAGAGG	Reverse primer for fragment E
Primer walking strategy, step 2—inner PCR		
3040	TGTGTTCTATGTCTCTCTCCTGGAGAGAGGTATAGAATG	Reverse primer for fragment A1 Paired with ODP 3100
3207	TTCCCTTTCATTCACGTCTGACTGC	Forward primer for fragment A2 Paired with ODP 3160
3184	TTAGAGAATTAGTGGCCCGCAGG	Reverse primer for fragment B1 Paired with ODP 3161
2934	CAATGGAACCTGGCG	Forward primer for fragment B2 Paired with ODP 3162
3189	AGAGTAATGGGGGTATCTGGTGC	Reverse primer for fragment D1 Paired with ODP 3165
2938	TCCTTGCAGGACCATGC	Forward primer for fragment D2 Paired with ODP 3024
3024	TCTCCATACACGTAGACT	Reverse primer for fragment D2 Paired with ODP 2938
3023	CACACCGTCAAGCACAGATT	Forward primer for fragment D3 Paired with ODP 3141
2944	GCGTGCCATGAAAAG	Reverse primer for fragment E1 Paired with ODP 2944
3039	TGATACTGACCATGGGCCCCAAATACCTTCC	Forward primer for fragment E2 Paired with ODP 3101

ODP, oligodeoxyribonucleotide primer; MGBNFQ, Minor groove binding non-fluorescent quencher

**Fig. 2:**
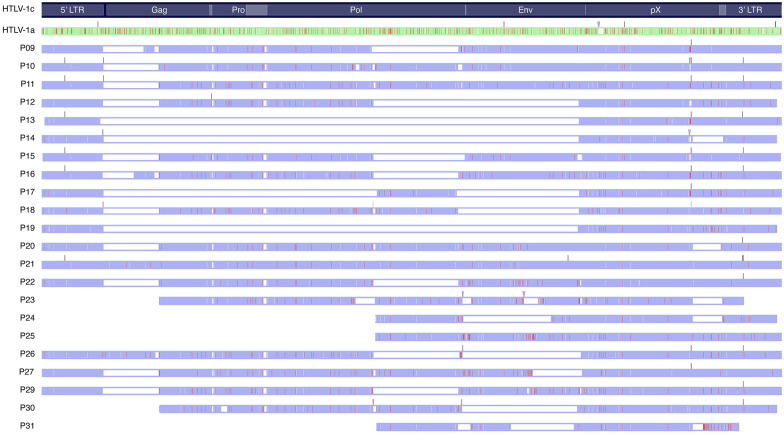
Proviral genomic deletions revealed from de novo assembly of Central Australian HTLV-1c genomic sequences. Linear map representation of de novo assembly of the 22 novel Central Australian HTLV-1c sequences (P9-31) in purple aligned to the consensus sequence. The consensus sequence of the Cosmopolitan HTLV-1a provirus generated from 11 previously published genomes (AB513134.1, AB979451.1, AF033817.1, AF042071.1, AF139170.1, HQ606137.1, HQ606138.1, KC807984.1, L03562.2, L36905.1, U19949.1), aligned in green. The major HTLV-1 genomic features are shown for the HTLV-1c consensus sequence (*Gag*, *Pro*, *Pol*, *Env*, *pX*, and LTRs). Red lines represent single nucleotide polymorphisms, white, represents gaps or deletions. Lines above the genome alignment represent insertions

De novo assembly was then performed with the subgenomic sequences to generate a consensus HTLV-1c proviral genome for each of the 22 HTLV-1c + donors, and subsequently aligned (GenBank: P09, PP596271; P10, PP596272; P11, PP596273; P12, PP596274; P13, PP596275; P14, PP596276; P15, PP596277; P16, PP596278; P17, PP596279; P18, PP596280; P19, PP596281; P20, PP596282; P21, PP596283; P22, PP596284; P23, PP596285; P24, PP596286; P25, PP596287; P26, PP596288; P28, PP596289; P29, PP596290; P30, PP596291; P31, PP596292)*.* The amplicons containing the 5′ LTR and structural genes (*gag-pol* and *env*) most frequently carried deletions. In contrast, amplicons containing the 3′regulatory region of the genome encoding products were largely intact and highly conserved (Fig. 2).

### HTLV-1c in Central Australia is genetically distinct from other HTLV-1 subtypes

The 22 proviral consensus sequences were then aligned with the four previously published Australian HTLV-1c genome sequences: Aus-CS, Aus-NR, Aus-DF, Aus-GM, and one Melanesian (MEL5) variant (Genbank: KF242506.1, JX891479.1, KF242505.1, JX891478.1 and L02534.1, respectively); 11 published HTLV-1a representative sequences available in Genbank (AB513134.1, AB979451.1, AF033817.1, AF042071.1, AF139170.1, HQ606137.1, HQ606138.1, KC807984.1, L03562.2, L36905.1, U19949.1) and the Simian T cell lymphotropic virus (STLV) strain considered to be the ancestral primate transmission strain isolated from naturally infected Celebes macaques (*Macaca tonkeana*) (STLV TE4, Z46900.1). The Sulawesi island habitat of the aforementioned STLV strain intersects with human migration paths proposed to have introduced HTLV-1c infection of humans into the Indo-Pacific and Oceania region [30].

**Fig. 3:**
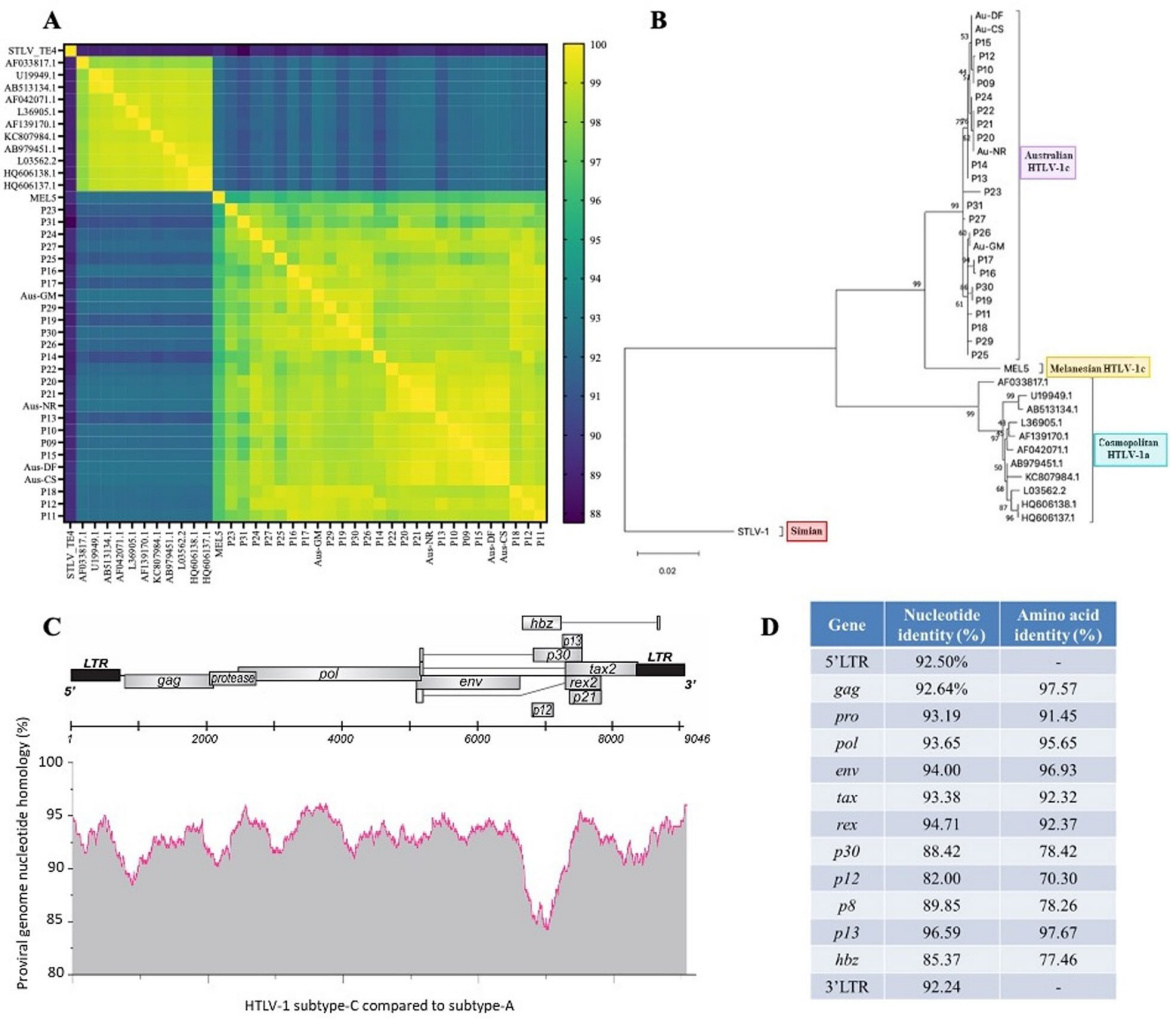
Australian HTLV-1c genome is highly divergent compared to HTLV-1a. **A** Pairwise comparisons of the newly assembled Central Australian HTLV-1c genomes (P9-31), the Melanesian HTLV-1c genome (MEL5), 4 previously available Australian HTLV-1c sequences (Aus-CS, Aus-DF, Aus-NR, Aus-GM), 11 HTLV-1a genomes (AB513134.1, AB979451.1, AF033817.1, AF042071.1, AF139170.1, HQ606137.1, HQ606138.1, KC807984.1, L03562.2, L36905.1, U19949.1), and one STLV genome (TE4). Sequence gaps were ignored. Colour indicates level of homology, with 100% shown in yellow, and 88% identity shown in dark blue. **B** Phylogenetic tree analysis on the concatenated 22 Central Australian HTLV-1c genomes generated in this work as well as the 4 HTLV-1c available sequences, shown in purple; the Melanesian HTLV-1c, shown in yellow; Simian, shown in red; and 11 Cosmopolitan HTLV-1a genomes shown in aqua. Outgroup rooting using the STLV-1 sequence was applied, and sequence gaps were ignored. The percentage of trees in which the associated taxa clustered together is shown above the branches. The branch lengths represent the number of substitutions per site. **C** Nucleotide homology between HTLV-1 subtype-C and subtype-A consensus sequences across the entire 9046 bp genome. **D** Percentage similarity of HTLV-1 LTRs and *orf* gene products between subtype-C and subtype-A consensus sequences. Nucleotide and translated amino acid identities are shown

Analysis of the novel Australian HTLV-1c assembled proviral genomes revealed a relatively high level of divergence within our cohort, with pairwise homology ranging between 96.61 and 99.91%, consistent with previous studies demonstrating subtype-C divergence [3]. The lowest homology between the Central Australian subtype-C genomes was observed in three participants: P23, P31 and P25. In fact, the newly assembled genomes reveal a higher level of subtype-C divergence than previously reported [3], where the pairwise homology between Aus-CS, Aus-NR, Aus-DF, and Aus-GM ranges between 98.87 and 100%. [31]. In comparison, the 11 previously published HTLV-1a genomes displayed a relatively high level of sequence identity (97.92–99.92%), consistent with the average low evolutionary rate estimate ranging from 2.98 to 5.04 × 10^−6^ substitutions/site/year measured for HTLV-1a in family chains of vertical transmission [31]. Pairwise comparisons between each subtype-A versus subtype-C genomes revealed 90.51–92.56% identity, with 6 donors from the Central Australian cohort (P31, P25, P17, P19, P14, P13) showing the highest divergence with HTLV-1a sequences. Homology between the Melanesian HTLV-1c genome and the novel Central Australian HTLV-1c genomes confirmed that the Central Australian subtype-C is distinct, with consistently lower pairwise identities (95.45–96.89%) than comparisons within Central Australia alone, consistent with previous reports [3]. The most divergent sequence identities, between 87.76 and 89.77%, were observed between the Sulawesi primate derived STLV genome and all HTLV-1a and -c sequences (Fig. 3A). Phylogenetic analyses of the 22 newly assembled Central Australian HTLV-1c genomes, with the aforementioned HTLV-1a, HTLV-1c and STLV genomes, confirmed the distinguishable features and divergence of the Central Australian HTLV-1c subtype (Fig. 3B). This is consistent with similar analyses of the four previously published subtype-C full-length genomes, which identified the emergence of two geographical-based clades of HTLV-1c in Central Australia: North (Aus-NR, Aus-DF, Aus-CF) and South (Aus-GM) over 9,000 years ago [3].

### Key genetic differences of HTLV-1 subtype-C occur in the 3′regulatory region of the proviral genome

The assembled HTLV-1c proviral genomic sequences from the 22 new participants were aligned to generate an Australian HTLV-1c consensus sequence of 9046 bp. Similarly, a consensus sequence was generated from the 11 HTLV-1a genomes analysed in this study. Comparison between the subtype-A and -C consensus genomes revealed an overall nucleotide identity of 92.5%, with homology ranging between 90 and 95% across most of the genome, spanning structural genes and proviral LTRs. However, strikingly, the lowest homology observed occurred near the 3′end of the viral genome encompassing regulatory genes encoded by the plus- and minus-RNA strand of the virus. A nucleotide identity as low as 83% occurred in the region overlapping the *orf-I* product p12 and *hbz* genes (Fig. 3C). Comparison of the nucleotide and translated amino acid (Table 3) sequences of each gene between HTLV-1a and -1c consensuses further revealed stark differences. The *orf-I* and *orf-II* genes encoding the p12-cleaved product p8 and p30, respectively, and antisense *hbz,* all displayed low nucleotide identity less than 90%, and similarly the amino acid homology of the encoded proteins was less than 80%. However, the HTLV-1c *orf-I* product p12 demonstrated the most significant divergence from subtype-A, in both the nucleotide and amino acid sequence (homology 82.00%, 70.30%, respectively) (Fig. 3D). Notably, a mutated start codon (AUG to ACG) of HTLV-1A *orf-I* (encoding the p12/p8 proteins) was present in 100% of the assembled genomes generated from each seropositive subject from Central Australia.

**Table 3:**
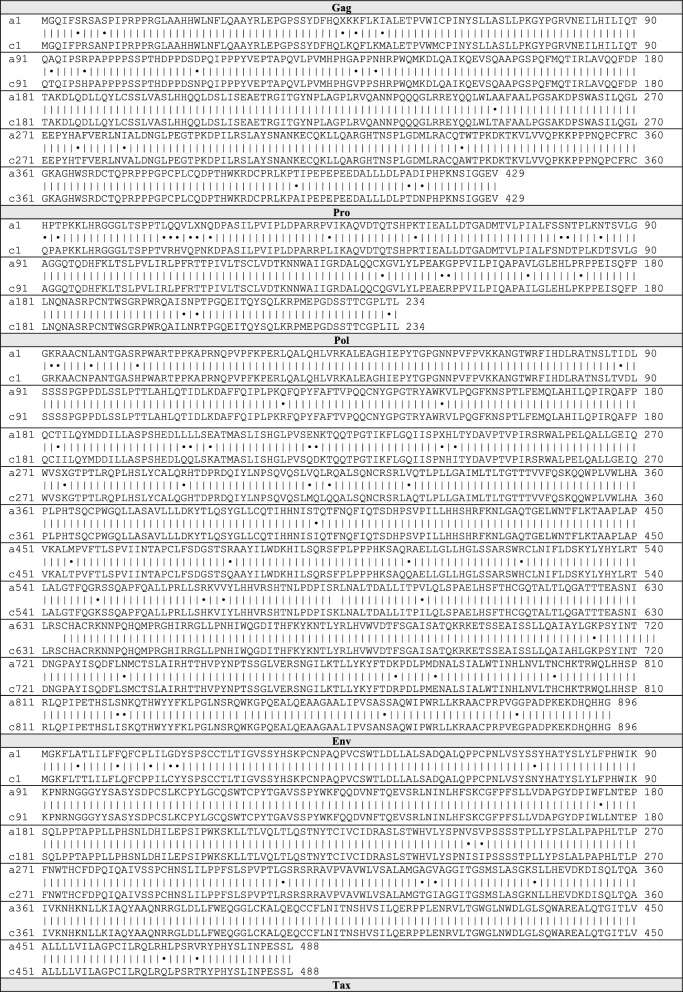

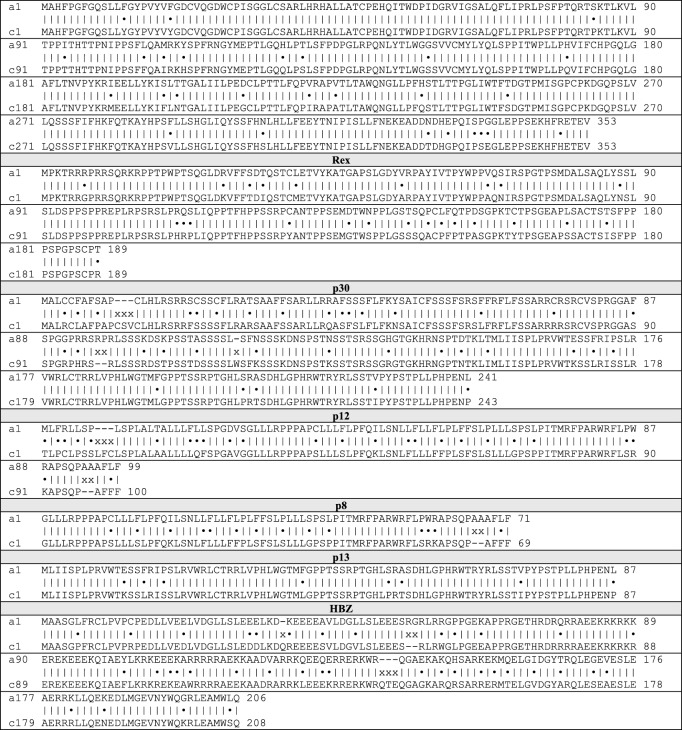
Comparison of HTLV-1 subtype-A and -C consensus protein sequences

Dot (•) represents amino acid difference, x represents gap in alignment

**Fig. 4:**
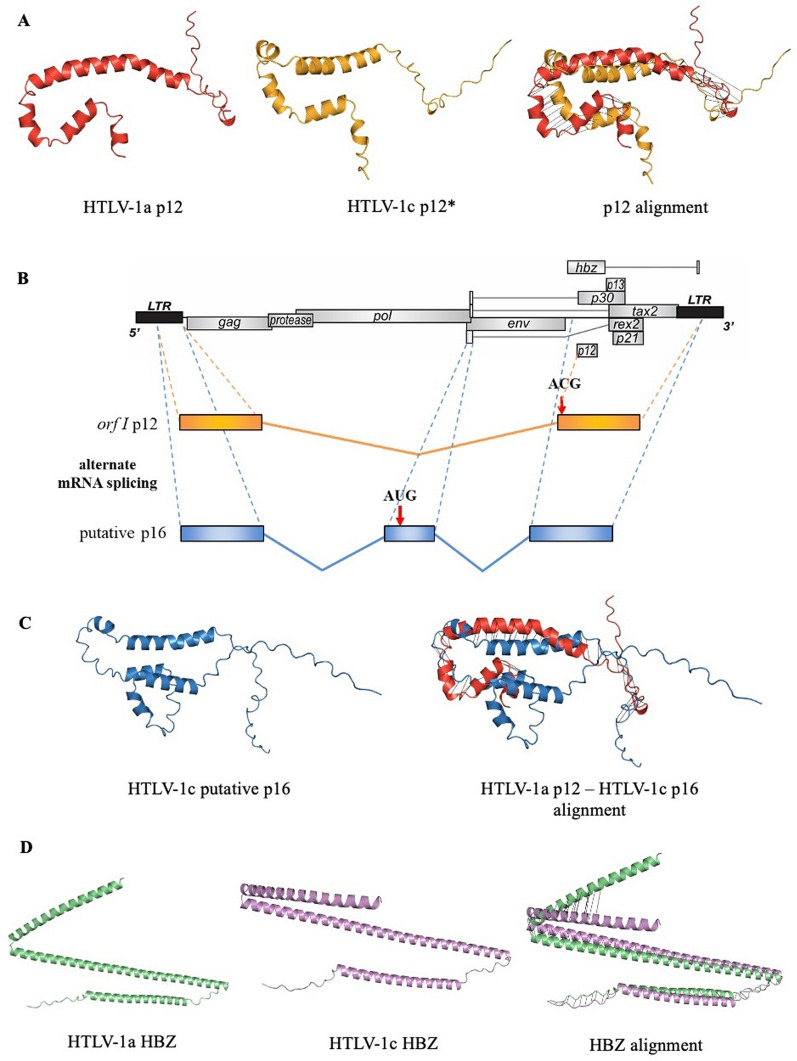
Key mutations in HTLV-1c *orf-I* p12 and *hbz* may facilitate alternate splicing and divergent protein structures. **A** Three-dimensional (3D) modelling of HTLV-1a (red) and HTLV-1c (yellow) *orf-I* product p12 based on the translated amino acid sequences of subtype-A (AB513134.1, AB979451.1, AF033817.1, AF042071.1, AF139170.1, HQ606137.1, HQ606138.1, KC807984.1, L03562.2, L36905.1, U19949.1) and subtype-C (P9-31, Aus-CF, Aus-DF, Aus-NR, Aus-GM, MEL5) consensus genomes. Star (*) represents an absence of p12 initiation codon, present in 100% of the sequences, due to a substitution AUG (Methionine) to ACG (Threonine). Black lines indicate regions of structural alignment. **B** Alternate mRNA splicing with *rex* exon 1, containing an intact start codon (AUG), and an alternate second acceptor site upstream of the equivalent HTLV-1a *orf-I* p12 site, is predicted to facilitate the expression of an HTLV-1c p16 product. **C** Comparative 3D modelling of HTLV-1a p12 (red) and putative HTLV-1c p16 product (blue). Black lines indicate regions of structural alignment.). **D** Alignment of HTLV-1a and HTLV-1c HBZ 3D protein structures, where regions of structural homology are indicated by black lines

Three-dimensional (3D) modelling of p12 of subtype-A with subtype-C revealed structural differences, in the event that post-transcriptional modifications could circumvent the mutated translation start codon in HTLV-1c (Fig. 4A). We postulated that HTLV-1c may use alternate splice sites for the pre-mRNA of *orf-I* to produce a doubly spliced transcript that incorporates an intact translation start codon in exon 1 of *rex* mRNA, which is conserved in HTLV-1c, creating a novel putative 16 KDa protein (p16) [26] (Fig. 4B). Comparative 3D modelling of the HTLV-1a p12 and the putative HTLV-1c p16 predict significant structural differences between these proteins, despite some small regions of structural relatedness (Fig. 4C). HBZ proteins are predicted to share structural relatedness between subtypes (Fig. 4D), although the amino acid sequences themselves have a high level of divergence (Table 3).

### Significantly higher retention of the 3′regulatory region than the structural region of the proviral genome in HTLV-1c infection

**Fig. 5:**
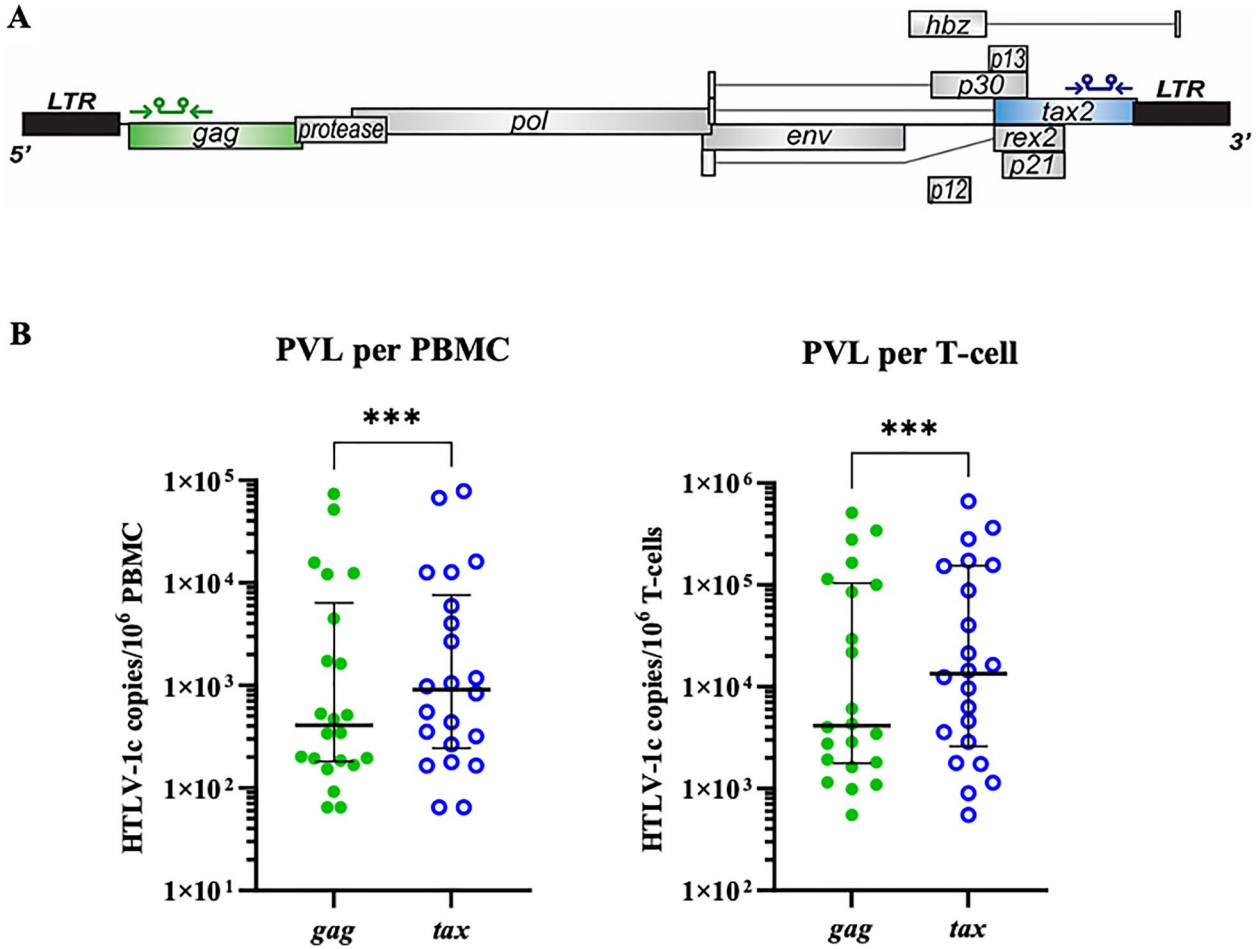
HTLV-1c *tax* is present at significantly higher levels than *gag* in PBMCs and T-cells. **A** Schematic of HTLV-1 provirus genome and positions of *gag* and *tax* primers and probes for ddPCR amplification. **B** Distributions of proviral load (PVL), HTLV-1c copies per 10^6^ PBMCs and per 10^6^ T-cells, detected by ddPCR targeting *gag* (green dots) and *tax* (blue hollow circles) gene regions, of 22 HTLV-1c positive subjects from Alice Springs Hospital cohort in Central Australia. Each data point represents a participant, and data shown for each participant are the mean values of quadruplet measurements. Median with interquartile range is shown by black lines. Statistical significance between matching data was assessed using Wilcoxon matched-pairs signed rank test (p, 0.0005, 0.0009, respectively)

To date, PVL appears to be the most important risk factor for HTLV-1-associated diseases as observed in HAM [19], HTLV-1 associated uveitis (HAU) [32], chronic lung diseases [10, 12], more extensive radiologically defined pulmonary injury [28] and death due to complications of bronchiectasis [12]. Through the application of droplet digital polymerase chain reaction (ddPCR), which quantifies DNA with high accuracy and greater sensitivity over a wide dynamic range compared to previous qPCR assays [33], we measured the PVL (HTLV-1c copies per 10^6^ cells) in the 22 participant gDNA samples using two sets of primer-probes targeting regions in the *gag* and *tax* genes (Fig. 5A). In parallel, ribonuclease P protein subunit P30 (RPP30, NCBI Gene ID: 10,556) was the human cellular housekeeping gene used as a reference. We further measured the PVL in T-cells (HTLV-1c copies per 10^6^ T-cells), by quantifying the absolute amount of unrearranged T-cell receptor (UTCR) in each sample, as previously described [33]. The *tax* PVL (median, 9.08 × 10^2^ copies per 10^6^ cells) was significantly higher (p, 0.0005) than *gag* PVL (median, 4.07 × 10^2^ copies per 10^6^ cells) in PBMCs when comparing the paired reads of each HTLV-1c + participant. Similarly, the *tax* PVL in T-cells (median, 1.34 × 10^4^ copies per 10^6^ T-cells) was greater than three times (p, 0.0009) what was detected using the *gag* PVL gene target (median, 4.16 × 10^3^ copies per 10^6^ T-cells) (Fig. 5B). This is consistent with the newly assembled Central Australian HTLV-1c genomes, where most donors presented an intact 3′-end of the proviral genome and deleted structural genes.

## Discussion

Unlike HIV-1, HTLV-1 does not deplete infected cells but may impart more subtle immune cell dysfunction for several decades [17, 34]. During the chronic phase of infection, the HTLV-1 PVL remains relatively stable over time [35] and correlates with disease outcome. A high HTLV-1c PVL was determined to be a major risk factor for bronchiectasis [28] and predicted death during follow up [12, 28] in the Central Australian setting. Our understanding of HTLV-1c genomic organization and pathogenesis is limited to five single previously published genomes. Hence the current study augments the database of subtype-C genomic structure by extending the molecular analysis to a large cohort of HTLV-1c infected individuals in Central Australia. Moreover, it is of great importance to accurately quantify HTLV-1 infected cells for clinical diagnostics and prognostics, as well as monitoring therapeutic applications, for which a greater understanding of the proviral genome sequence is essential.

While the overall genomic homology of HTLV-1c with HTLV-1a is 92.5%, we identified several key differences at the nucleotide level which could impact viral gene expression. The HTLV-1c 3′regulatory region encoding *tax*, *hbz*, and *orf-I and orf-II* products, that are central for viral transcription [22, 36], cellular transformation [21, 37, 38], transmission [26, 39] and hijacking host immunity [39], is consistently retained in all participants. However, the observed increased genomic divergence from subtype-A in the subtype-C regulatory region near the 3′end of the genome could profoundly impact viral pathogenesis. Critically, the HTLV-1a *orf-I* translation start codon for p12 was absent in 100% of the HTLV-1c-infected subjects, which could affect viral transmission and immune evasion [25, 26]. However, it is possible that HTLV-1c has overcome the absence of the *orf-I* AUG codon by an alternatively spliced mRNA that provides an initiation of translation codon to *orf-I* p12, to produce a p16 product [26]. Conversely, other transmission mechanisms not reliant on an intact p12 protein, such as de novo infection and clonal expansion of infected T-cells, may be operating in HTLV-1c infection. Moreover, these genomic differences may impact the expression of the overlapping antisense gene *hbz*, that is known to contribute to viral pathogenesis, proliferation and persistence through both its functional RNA and the encoded protein [24]. Nucleotide indels or mutations can impact the 2- and 3-dimensional folding and epigenetic modifications of both RNA and protein, and thus may alter, augment or decrease the functional capacity of such entities. Future studies investigating the provirus structure and viral RNA expression are needed to understand how the genomic divergence of HTLV-1c in Central Australia impacts pathogenesis, persistence, and associated disease progression, particularly bronchiectasis.

Importantly, this study demonstrated that with the high frequency of provirus containing structural gene deletions, the confirmatory diagnostic qPCR test targeting the *gag* region initially used in Australia for HTLV-1, can be improved with targeting other more conserved regions of the HTLV-1c genome, such as *tax* exon 2. This will lead to greater sensitivity of detection when PVL is low, and greater accuracy in measuring the number of infected cells, when proviruses may have deleted structural genes.

A limitation of this study to assemble whole HTLV-1c proviral genomes from overlapping contigs, lies in the characterisation of exclusively expected high molecular weight bands achieved in the subgenomic PCR amplifications. Further in-depth investigation of defective proviruses by sequencing smaller molecular weight PCR products, or long-read sequencing approaches, will provide greater insights into the ability of infected cells carrying defective or intact proviruses to participate in viral transmission and expression of pathogenic viral products. In addition, it would be valuable to compare the provirus landscape by single-molecule approaches of HTLV-1c with HTLV-1a, which reports the presence of proviruses with 5′ and internal deletions in participants with ATL, HAM and asymptomatic carriers by fragmented DNA-capture-seq [40]. Furthermore, with proviral loads in our HTLV-1c cohort as high as 10% in some individuals, it would be valuable to investigate the clonality of these infected cells [41], and whether there are multiple copies of provirus present, defective or intact, inside the same cell [42].

While this study focuses on the HTLV-1c genomic sequence differences to HTLV-1a and the potential pathogenetic implications, future studies should also address host and environmental factors that may impact infection and pathogenesis in Central Australia. Host genetics, co-morbidities and co-infections, and transmission routes, must all be considered when investigating the complex host-virus interplay, that results in a frequent association of HTLV-1c infection with bronchiectasis [10, 11] and blood stream co-infections [14], as well as non-communicable diseases like chronic kidney disease [15, 16].

In conclusion, the present study highlights and expands the understanding of the genomic divergence of HTLV-1c in Central Australia and gives rise to the hypothesis that differences in *orf-I* and *hbz* expression may have pathogenic consequences in HTLV-1c infection, which is more commonly associated with bronchiectasis and blood stream co-infections. Further studies are needed to investigate the impacts these genetic differences have on viral RNA and protein functions, and a deeper characterisation of the proviral landscape in vivo, to better understand the nuances of HTLV-1c pathogenesis, and the factors that lead to associated pulmonary disease development.

## Materials and methods

### HTLV-1 serological and clinical analyses

Diagnosis of HTLV-1 serostatus was based on the detection of specific anti-HTLV-1 antibodies in serum by enzyme immunoassay (EIA) (Murex HTLVI + II; DiaSorin, Saluggia, Italy) and the Serodia HTLV-I particle agglutination assay (Fujirebio, Tokyo, Japan) performed by the National Reference Laboratory, Victoria, Melbourne, Australia. Demographic and clinical characteristic differences between serostatus groups were analysed using Mann–Whitney U test for continuous variables, and Fisher’s exact test for categorical variables, and statistical significance determined with p < 0.05.

### Genomic DNA extraction

Thirty-seven deidentified frozen peripheral blood buffy coats from Alice Springs Hospital obtained with ethics consent, consisting of 22 HTLV-1c + , and 15 HTLV-1c-, were sent to The Doherty Institute for Infection and Immunity, at The University of Melbourne. Genomic DNA (gDNA) was extracted using GenElute™ Blood Genomic DNA Kit (Sigma-Aldrich) according to manufacturer’s instructions and eluted in EB buffer (Sigma-Aldrich). Quantification and purity of the isolated gDNA was measured by UV spectrophotometry (Nanodrop Technologies, Wilmington, CA).

### HTLV-1 Proviral Load Measurement

The HTLV-1 proviral load was measured as previously described [33] by droplet digital polymerase chain reaction (ddPCR) using 100 ng of gDNA mixed with forward and reverse primers (each 900 nM) targeting either HTLV-1c *gag* or *tax*, and the corresponding TaqMan probe (5ʹ-FAM, MGBNFQ-3ʹ, 250 nM, ThermoFisher Scientific) (Table 2). ddPCR was performed using ddPCR Supermix for probes (no dUTP, Bio-Rad Laboratories, Hercules, CA) and included the reference gene RPP30 (Ribonuclease P/MRP subunit P30, HEX, dHsaCPE5038241, Bio-Rad). All samples were run in quadruplet, and the reported HTLV-1c PVL is the mean of the 4 measurements. HTLV-1c negative gDNA was used for negative controls. The T-cell population was determined by measuring the loss of the Dβ1-Jβ1.1 intergenic sequences, as previously described [33]. The ddPCR limit of detection for HTLV-1 PVL was determined to be 65 copies per 10^6^ PBMCs and 98 copies per 10^6^ T-cells. Statistical significance for matched *gag* and *tax* PVL data was assessed using Wilcoxon matched-pairs signed rank test.

### Genome walking PCR

The sequencing of HTLV-1c proviral genomes from genomic DNA samples isolated from 22 participants was completed based on a genome walking strategy, as previously described [3]. The overlapping primers used in the nested PCR are reported in Table 2. HTLV-1c negative gDNA was used for negative controls. PCR reactions were completed using Phusion High-Fidelity DNA Polymerase (ThermoFisher Scientific) and 50-200 ng of gDNA following the manufacturer’s instructions. Cycle parameters were as follows: enzymatic activation for 5 min at 98 °C; 35 cycles of (denaturation for 10 s at 98 °C, annealing for 30 s at 60–64 °C, and extension for 30 s/kb at 72 °C); followed by a final extension for 10 min at 72 °C; and infinite hold at 10 °C. PCR products were electrophoresed through a 1% agarose gel in the presence of GelRed (Biotium) in parallel with 1 Kb Plus DNA ladder (ThermoFisher Scientific) in 1 × TAE buffer at 100 V. DNA was purified from excised gel bands with the NucleoSpin Gel and PCR Clean-up Kit (Macherey–Nagel) as per the manufacturer’s instructions. The PCR products were then sequenced using the BigDye Terminator v3.1 Cycle Sequencing Kit (ThermoFisher Scientific) following manufacturer’s instructions with DNA and primers added at 100 ng/kb of template and 5 µM, respectively. Sequencing reactions were purified using the DyeEx 2.0 Spin Kit (Qiagen) as per manufacturer’s instructions then sequenced by the Centre for Translational Pathology (Department of Pathology, University of Melbourne).

### Genomic sequence analysis

Construction of HTLV-1c genomic consensus sequence: All sequences obtained were checked against the electropherograms and aligned via ClustalW sequence alignment algorithm using Seaview v4.5.4 [43]. Multiple sequence alignments of assembled contigs, HTLV-1a and HTLV-1a, and STLV sequences were performed using Clustal Omega [44]. *Phylogenetic analysis.* A phylogenetic tree rooted to the STLV-1 sequence was generated using the Maximum Likelihood method and the Tamura-Nei model in MEGA11 [45, 46]. The Tamura-Nei model was selected based on Bayesian Information Criterion (BIC) scores to best describe the substitution pattern. The tree with the highest log likelihood (-3597.65) is presented. The percentage of trees in which the associated taxa clustered together is shown above the branches. Initial tree(s) for the heuristic search were obtained automatically by applying Neighbor-Join and BioNJ algorithms to a matrix of pairwise distances estimated using the Tamura-Nei model, and then selecting the topology with superior log likelihood value. A discrete Gamma distribution was used to model evolutionary rate differences among sites [5 categories (+ G, parameter = 0.5462)]. The tree was drawn to scale, with branch lengths measured in the number of substitutions per site. This analysis involved 39 nucleotide sequences. Outgroup rooting using STLV-1 was applied. All positions containing gaps and missing data were eliminated (complete deletion option). There were a total of 1443 positions in the final dataset. *Genomic percentage identity.* Percentage homology of each nucleotide across the consensus sequences comparing of subtype-A and -C were determined in Geneious Prime v2023.2, with parameter of ignoring gaps, and visualised in GraphPad prism v10.1.2 with 0th order curve smoothing to 200 neighbouring nucleotides. *Gene percentage identity.* Each gene was analysed individually by comparing subtype-A and -C consensus sequences using BLAST [47]. *Three-dimensional protein structures*. Amino acid sequences of the consensus HTLV-1a p12, HTLV-1c p12, putative HTLV-1c p16, HTLV-1a HBZ and HTLV-1c HBZ products were used for the generation of protein data bank (PDB) structures with Alphafold2 [48, 49]. The first ranked structure was chosen for each protein, and three-dimensional modelling and alignments was performed using Pymol v2.5.7 [50].

## Data Availability

The proviral genomic DNA datasets generated during the current study are available in the GenBank repository (accession nos. PP596271, PP596272, PP596273, PP596274, PP596275, PP596276, PP596277, PP596278, PP596279, PP596280, PP596281, PP596282, PP596283, PP596284, PP596285, PP596286, PP596287, PP596288, PP596289, PP596290, PP596291, PP596292).
